# Behavior Change Theories and Models Within Health Belief Model Research: A Five-Decade Holistic Bibliometric Analysis

**DOI:** 10.7759/cureus.63143

**Published:** 2024-06-25

**Authors:** Ahmed S Alamer

**Affiliations:** 1 Health Education and Promotion, Jazan University, Jazan, SAU

**Keywords:** theoretical framework, health behavior, public health, bibliometric analysis, health belief model

## Abstract

The health belief model (HBM) has gained significant scholarly attention over the past five decades. This study aims to provide a comprehensive bibliometric analysis of the HBM research landscape to reveal its evolving trends and impact. The analysis utilized data from the Scopus database to explore publication patterns, influential sources and researchers, international collaborations, and thematic trends in the HBM-related literature. The findings demonstrate a substantial growth in HBM publications, with a peak of more than 11% in 2022-2023. The United States, Iran, China, the United Kingdom, and Australia are the most productive countries, and prominent HBM researchers include Lau JTF, Champion VL, and Jeihooni AK. Thematic analysis reveals a shift from broader topics of HBM to more specific areas, such as COVID-19, health behavior, and digital health interventions. The current study highlights the sustained and growing interest in HBM, its diverse applications, and the collaborative efforts of researchers worldwide to develop and refine this influential theoretical framework in public health and behavioral science.

## Introduction and background

Behavior change theories and models provide a comprehensive framework for understanding the complex process of how and why individuals or populations change their behaviors [1,2]. The Transtheoretical Model, also known as the Stages of Change, proposes that behavior change occurs through six stages: precontemplation, contemplation, preparation, action, maintenance, and termination, with individuals moving through these stages at their own pace [3]. The Theory of Planned Behavior suggests that an individual's intention to perform a behavior is the key determinant, influenced by their attitudes, subjective norms, and perceived behavioral control [4]. Social Cognitive Theory emphasizes the dynamic and reciprocal interaction between the individual, their behavior, and the environment, including key concepts such as self-efficacy, observational learning, and reinforcement [5]. The Ecological Model considers multiple levels of influence on behavior, including intrapersonal, interpersonal, organizational, community, and policy factors [6]. These theories and models provide a foundation for understanding behavior change and informing the design of effective interventions to promote healthier behaviors, and researchers and practitioners often draw on multiple models to guide their approaches [5,7].

The health belief model (HBM) is a widely used theoretical framework for understanding and predicting health-related behaviors. The model posits that an individual's likelihood of engaging in a health-promoting behavior is determined by their perceived susceptibility to a health threat, perceived severity of the health threat, perceived benefits of taking action, and perceived barriers to taking action. In the context of health promotion and health education, HBM can be a valuable tool for designing and implementing effective interventions [8-10].

Health education efforts can focus on increasing individuals' perceived susceptibility to health risks or conditions, such as educating people about their risk of developing type 2 diabetes based on their lifestyle factors and family history, to motivate them to adopt preventive behaviors [10,11]. Highlighting the potential serious consequences of a health condition, such as long-term complications of untreated hypertension, can help people recognize the severity of the threat and the importance of taking action. Health promotion programs can emphasize the benefits of adopting healthy behaviors, such as the positive impact of regular exercise on cardiovascular health or the reduced risk of certain cancers through early detection. Health education initiatives can address perceived barriers to engaging in health-promoting behaviors, such as the cost of healthy foods, the lack of access to healthcare, or time constraints, to make it easier for individuals to take action. Health promotion efforts can also provide "cues to action" that prompt individuals to engage in recommended behaviors, such as reminder messages, media campaigns, or requests from healthcare providers [1,12,13].

By applying the HBM, health professionals can develop targeted interventions that address the specific perceptions and beliefs that influence an individual or a community's health-related behaviors [7]. This can involve tailoring educational materials, implementing behavior change strategies, and using various communication channels to reach and engage the target population effectively [9]. HBM has been widely used in multiple health-related contexts, including promoting vaccination uptake, increasing cancer screening rates, encouraging adherence to chronic disease management regimens, and promoting healthy lifestyle behaviors such as cessation of smoking, physical activity, and healthy eating. In general, HBM provides a comprehensive framework for understanding the psychological factors that influence health-related behaviors and its application in health promotion and health education can contribute to the development of more effective and impactful interventions [1,14,15].

The HBM was chosen as the focus of this article due to its widespread use and applicability in health behavior change. The HBM encompasses multiple constructs, providing a comprehensive framework for understanding behavior change. Its extensive literature allows for in-depth analysis of publication trends, citation patterns, and collaborative networks. By focusing on the HBM, this study aims to advance the integration of behavior change theories and models, inform research priorities, and foster interdisciplinary collaborations for more effective health behavior change interventions.

This study aims to conduct a comprehensive scientometric analysis of behavior change theories and models within the HBM context. The existing literature has focused primarily on individual applications, but a holistic exploration of the evolving landscape is lacking. This scientometric investigation seeks to analyze publication trends, citation patterns, and influential factors; examine collaborative networks and intellectual structures highlighting interconnections between behavior change frameworks; uncover thematic evolution and emerging research directions; and provide recommendations to advance the integration of behavior change theories and models within the HBM framework, informing future research priorities and interdisciplinary collaborations for more comprehensive health behavior change interventions.

## Review

Materials and methods 

Database Selection

Although other databases such as WOS and PubMed could also be considered for bibliographic data extraction, Scopus was selected as the primary source for this study due to its broader coverage, multidisciplinary scope, enhanced citation analysis capabilities, and overall reliability and timeliness of the data [16]. The use of Scopus ensures a comprehensive and high-quality data set for the proposed scientometric exploration of theories and models of behavior change within the HBM research domain.

Search Strategy and Data Mining

The search query used to extract the bibliographic data for this study was (TITLE-ABS-KEY("Health belief model")), with no time or language limitations, and all types of documents, such as articles, reviews, conference papers, and others, were included. The data, consisting of 5934 records, were extracted on 1.04.2024 in two formats (CSV and BibTex), which includes a comprehensive set of metadata, including the year of publication, title, authors, keywords, corresponding country, abstracts, citations, references, source, and document type. The HBM research landscape is dominated by journal articles, which represent 88.98% of total document output, followed by reviews (5.71%), conference papers (1.97%), and book chapters (1.57%). The remaining 1.77% comprised other types of documents, such as editorials, letters, and notes, indicating that the field relies heavily on peer-reviewed publications to disseminate its theoretical developments and empirical findings across a variety of public health and behavioral science disciplines.

Data Analysis and Mapping

The Bibliometrix R package (4.2.2) and VOSviewer software (1.6.20) were used to analyze the data [17,18]. The analyses included descriptive statistics to provide an overview of the research trends and impact, temporal analysis to examine publication and citation patterns over time, collaboration analysis to visualize coauthorship networks and uncover intellectual structures, and keyword analysis to explore the thematic evolution and emerging research directions [19,20]. These applications were also used to create visual representations, including co-authorship networks to identify key collaborating countries and co-occurrence networks of keywords to map thematic landscapes and emerging research themes within the HBM research domain [17,18]. The combined use of these scientometric tools and techniques allowed a comprehensive exploration of the interconnections between behavior change theories and models, as well as the identification of prominent research trends, influential factors, and areas for future investigation within the context of HBM. There are two standard weight attributes, referred to as the links attribute and the total link strength (TLS) attribute. For a given item, the links and TLS attributes indicate, respectively, the number of links of an item with other items and the total strength of the links of an item with other items [17].

Results

**Figure 1:**
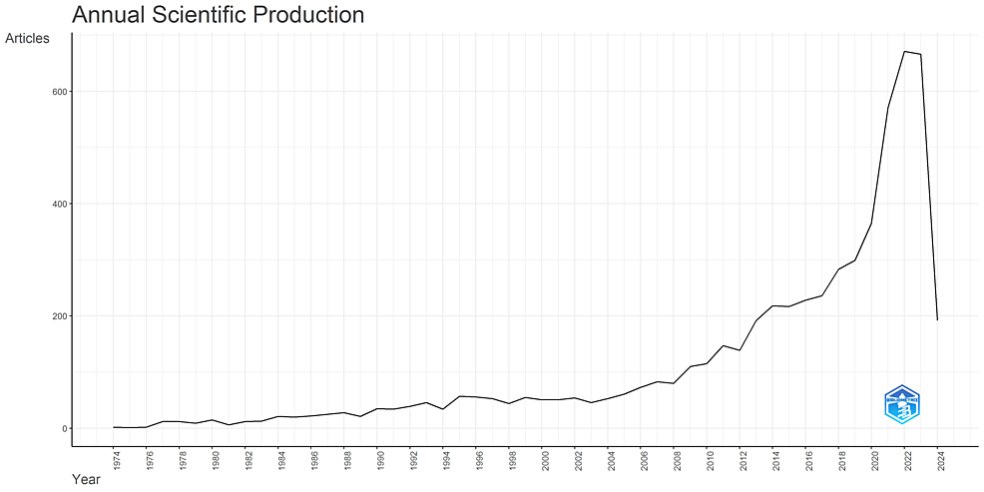
The trends in the annual publication of HBM research over time. The y-axis shows the number of articles published, while the x-axis depicts the years since the first HBM-related article was published. The graph demonstrates a clear upward trend, indicating a growing interest and scholarly focus on the HBM, with the publication volume rising substantially over the course of the time period. HBM, health belief model

The trends in the annual publication of HBM research over time are shown in Figure 1. The data span from 1974, the year the first HBM-related article was published, up to March 2024, and demonstrates a steady increase in the number of HBM publications over the years. In the early years, the annual publication count was relatively low, ranging from one (0.02%) to 35 (0.60%) articles, but the 1990s saw a pickup in activity, with the annual number of articles ranging from 34 (0.58%) to 57 (0.97%). From the 2000s onward, there has been a significant increase in HBM research output, with the number of annual publications consistently exceeding 100 and reaching a peak of 671 (11.42%) in 2022, while the percentage of total HBM publications has also risen substantially, from less than 1% in the 1970s and 1980s to more than 11% in 2022 and 2023, clearly indicating the growing interest and scholarly focus on HBM over the past several decades.

**Figure 2:**
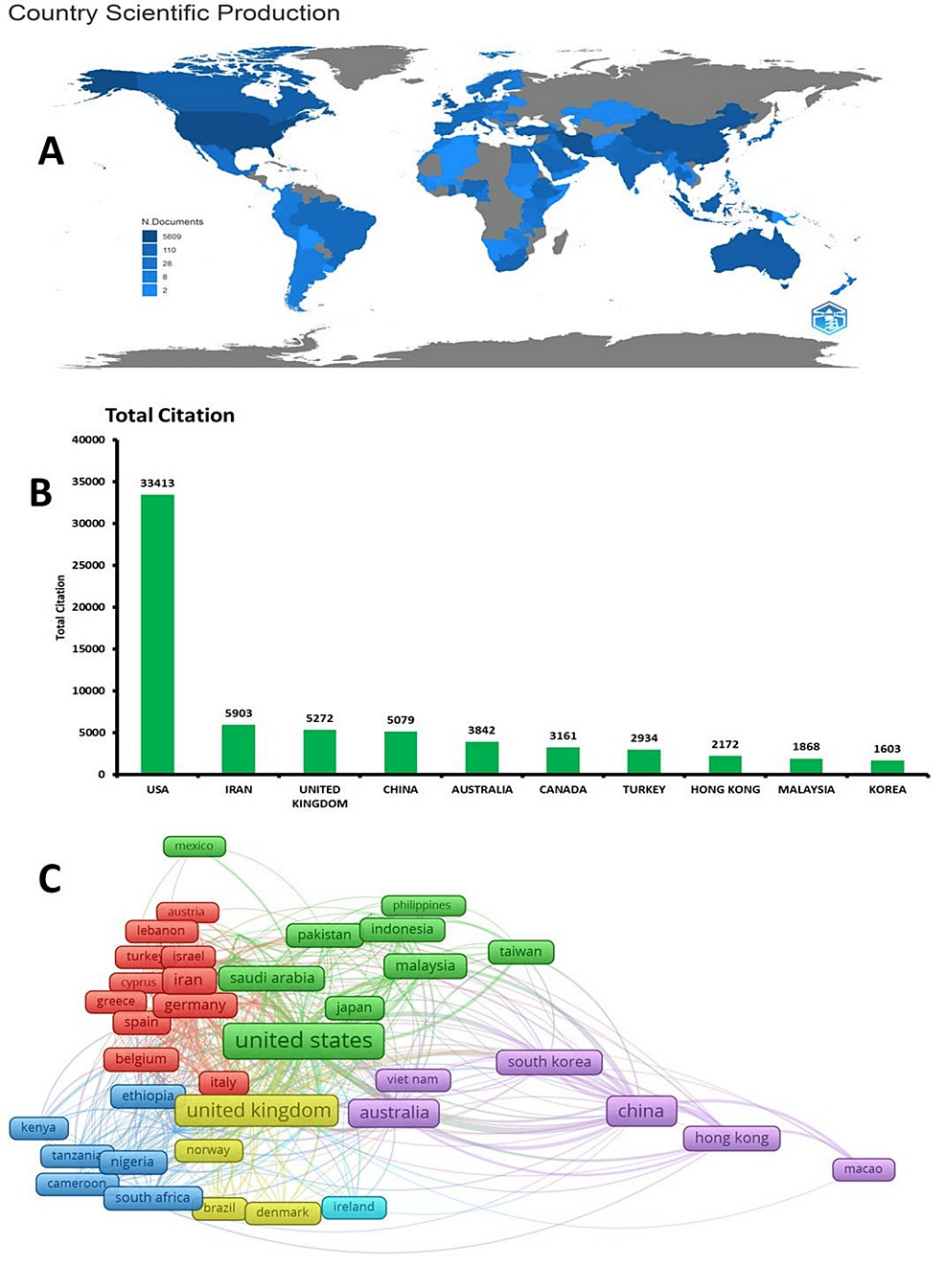
A: The global map shows the geographical distribution of HBM research production; B: Most cited countries in HBM research; C: Most collaborative countries. Darker blue areas in A indicate higher levels of production. The United States, Iran, and China are the dominant producers. The United States, the United Kingdom, China, Australia, and Canada are emerging as the most influential. This figure was generated using the VOSviewer application and a CVS data file. HBM, health belief model

The most productive sources of HBM research include the International Journal of Environmental Research and Public Health with 108 documents, BMC Public Health with 106 documents, PLOS One with 92 documents, Asian Pacific Journal of Cancer Prevention with 85 documents, and Vaccines with 79 documents. The most productive affiliations are the University of Michigan, Ann Arbor, with 73 documents; the Chinese University of Hong Kong, with 72 documents; Tehran University of Medical Sciences, with 66 documents; Shiraz University of Medical Sciences, with 63 documents; and Shahid Beheshti, University of Medical Sciences, with 59 documents. The United States leads the top 10 countries (Figure 2A) in terms of the number of documents related to HBM research with 5609 documents, followed by Iran with 1965 documents, China with 1364 documents, the United Kingdom with 665 documents, Australia with 614 documents, Canada with 508 documents, Malaysia with 396 documents, Turkey with 386 documents, Indonesia with 330 documents, and South Korea with 284 documents, reflecting the global prominence and diverse international contributions to the development and application of HBM in a range of public health and behavioral science domains. In terms of total citations (Figure 2B), the countries with the highest number of citations are the United States with 33413 citations, Iran with 5903 citations, the United Kingdom with 5272 citations, China with 5079 citations, and Australia with 3842 citations, highlighting the global impact and reach of HBM research. The top-publishing scholars in HBM research are Lau JTF (Hong Kong), followed by Champion VL (United States), and Jeihooni AK (Iran).

Based on the information (Figure 2C) provided on the TLS of different countries in the data given, the countries with the most collaborative are the United States (TLS=571), the United Kingdom (TLS=326), China (TLS=241), Australia (TLS=170), and Canada (TLS=156). The shapes in Figure 2C represent the TLS of each country, indicating the strength of scientific collaboration, with larger shapes suggesting more collaborative activity between researchers from that country and their international counterparts. The data clearly show that the United States is the most cooperative, followed by the other four countries, which also have relatively high TLS values, indicating strong international research collaborations in HBM research.

Table 1 presents the top 10 most cited documents in HBM research. The most cited document is "The Health Belief Model: A Decade Later," published in 1984 with 5701 total citations and a citation average of 139.05, indicating its significant and lasting impact on the field. The second and third most cited documents are "Social Learning Theory and the Health Belief Model" (1988) and "Historical Origins of the Health Belief Model" (1974), both published in Health Education & Behavior, highlighting the importance of the theoretical foundations and development of the HBM, with 3502 and 3426 citations, respectively, and citation averages of 94.65 and 67.18. The table also includes more recent influential works, such as "The role of behavioral science theory in development and implementation of public health interventions" (2010) and "A meta-analysis of the effectiveness of HBM variables in predicting behavior" (2010), which have received 1396 and 868 citations, with citation averages of 93.07 and 57.87, respectively, demonstrating the continuing relevance and application of the HBM in public health and behavioral research, while the remaining documents cover a range of topics, from preventive health behavior and adolescent decision-making to medication compliance and health-protective behavior theories, underscoring the versatility and broad applicability of the HBM across various health-related domains.

**Table 1:** Top-cited documents in HBM research. [1,10,14,15,21-26] CA, citation average; HBM, health belief model

Rank	Title	Year	Source	Citations	CA
1st	The health belief model: a decade later	1984	Health Education & Behavior	5701	139.05
2nd	Social learning theory and the health belief model	1988	Health Education & Behavior	3502	94.65
3rd	Historical origins of the health belief model	1974	Health Education & Behavior	3426	67.18
4th	The health belief model and preventive health behavior	1977	Health Education & Behavior	1900	39.58
5th	The role of behavioral science theory in development and implementation of public health interventions	2010	Annual Review of Public Health	1396	93.07
6th	Risk and rationality in adolescent decision making: implications for theory, practice, and public policy	2006	Psychological Science in the Public Interest	928	48.84
7th	A meta-analysis of the effectiveness of health belief model variables in predicting behavior	2010	Health Communication	868	57.87
8th	Determinants of medication compliance in schizophrenia: empirical and clinical findings	1997	Schizophrenia Bulletin	833	29.75
9th	Testing four competing theories of health-protective behavior	1993	Health Psychology	722	22.56
10th	Self-efficacy in the adoption and maintenance of health behaviors: theoretical approaches and a new model	2014	In Self-Efficacy: Thought Control of Action	689	62.64

**Figure 3:**
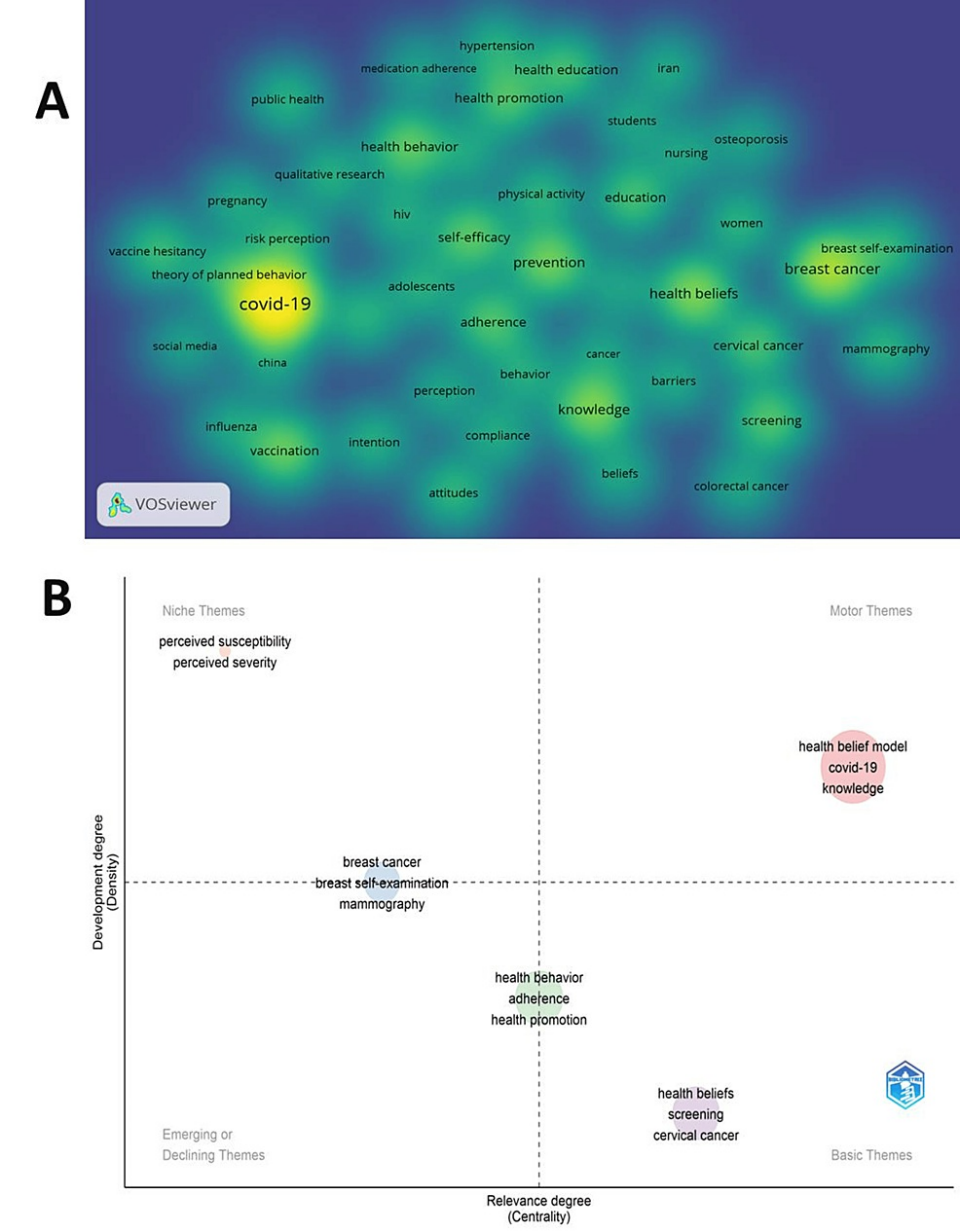
A: International collaboration; B: Thematic map of HBM research. A: This figure visualizes the TLS of different countries in HBM research, an indicator of their scientific collaboration. The size of each country's shape is proportional to its TLS value, with larger shapes denoting stronger international collaboration. This figure was generated using the VOSviewer application and a CVS data file. B: It was generated using the Bibliometrix application and a BibTex data file. The map is divided into four quadrants based on the centrality and density of the research topics, indicating their importance and development within the HBM research domain. This visual representation offers insights into the core, specialized, emerging, and declining areas of focus, guiding researchers and practitioners toward the most impactful and well-developed topics, as well as potential future research directions in HBM research. TLS, total link strength

The keywords used most frequently by the authors in HBM research (Figure 3A) reflect the central focus on the HBM itself, with 2160 occurrences of this keyword. Other prominent keywords include those related to specific health issues, such as "covid-19" (497 occurrences), "breast cancer" (217 occurrences), and "cervical cancer" (100 occurrences), as well as constructs like "health beliefs" (161 occurrences), "knowledge" (160 occurrences), "self-efficacy" (123 occurrences), and "adherence" (109 occurrences). The prevalence of keywords associated with preventive health behaviors, including "prevention" (145 occurrences), "vaccination" (134 occurrences), "health behavior" (125 occurrences), and "screening" (112 occurrences), suggests a strong emphasis on understanding and promoting various health-related behaviors within the HBM research landscape. Additionally, keywords such as "health promotion" (106 occurrences) and "education" (100 occurrences) indicate the diverse applications of HBM and the role of educational interventions in this field.

Figure 3B presents a conceptual mapping of HBM research based on Callon centrality and Callon density metrics, categorizing the themes into four quadrants. The "HBM" is identified as a motor theme, with high centrality and density, indicating that it is a core and well-developed area of research. "Breast cancer" and "perceived susceptibility" are classified as niche themes, suggesting that they are specialized areas of less central importance. The themes of "health behavior" and "health beliefs" are considered basic themes, reflecting their foundational nature within the HBM research domain. This analysis provides insight into the relative importance and interconnectedness of various topics related to HBM, guiding future research directions and priorities.

**Figure 4:**
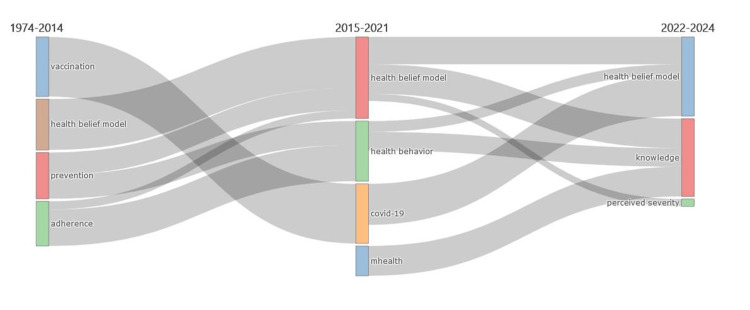
Evolution of thematic foci in HBM research over the past five decades. This figure was generated using the Bibliometrix application and a BibTex data file. HBM, health belief model

Figure 4 presents the thematic evolution of HBM research over the past five decades, from 1974-2014 to 2015-2021 and the most recent period of 2022-2024. The key themes and their transitions show a shift from broader topics such as adherence, prevention, and HBM itself in the earlier period to more specific areas such as COVID-19, health behavior, and mHealth in the more recent years. The most recent period (2022-2024) further refines the focus toward the core constructs of the HBM, such as knowledge and perceived severity, within the context of emerging areas like COVID-19 and digital health interventions.

**Figure 5:**
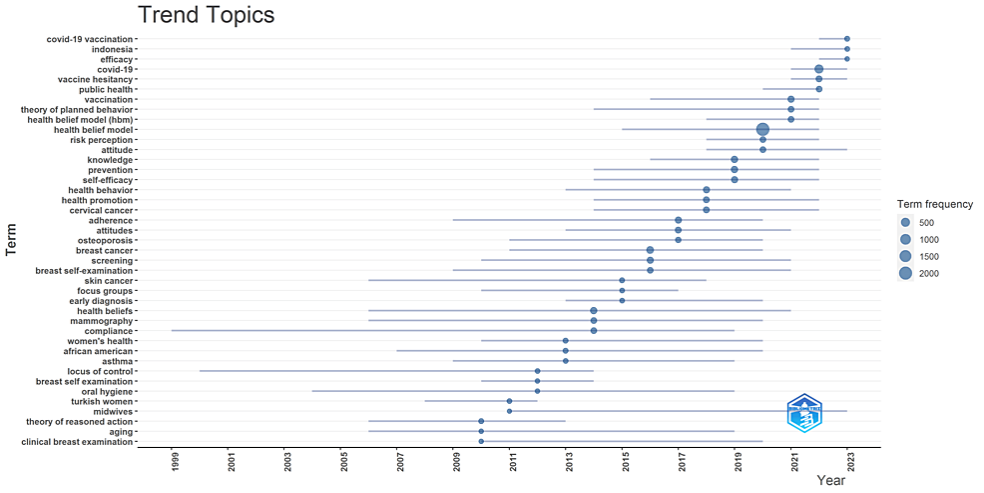
Trending topics in HBM research over the past 15 years. The horizontal lines represent the lifespan of each topic, while the circles correspond to the frequency of occurrence. This visualization allows for the identification of emerging themes. This figure was generated using the Bibliometrix application and a BibTex data file.

Trending topics in HBM research over the past 15 years are shown in Figure 5. The horizontal lines represent the lifespan of each topic, while the circles correspond to the frequency of occurrence. The emerging themes are "COVID-19 vaccination," "efficacy," "vaccine hesitancy," "Indonesia," "public health," "risk perception," "attitude," "knowledge," "health promotion," "cervical cancer," "prevention," and "self-efficacy."

Discussion

**Figure 6:**
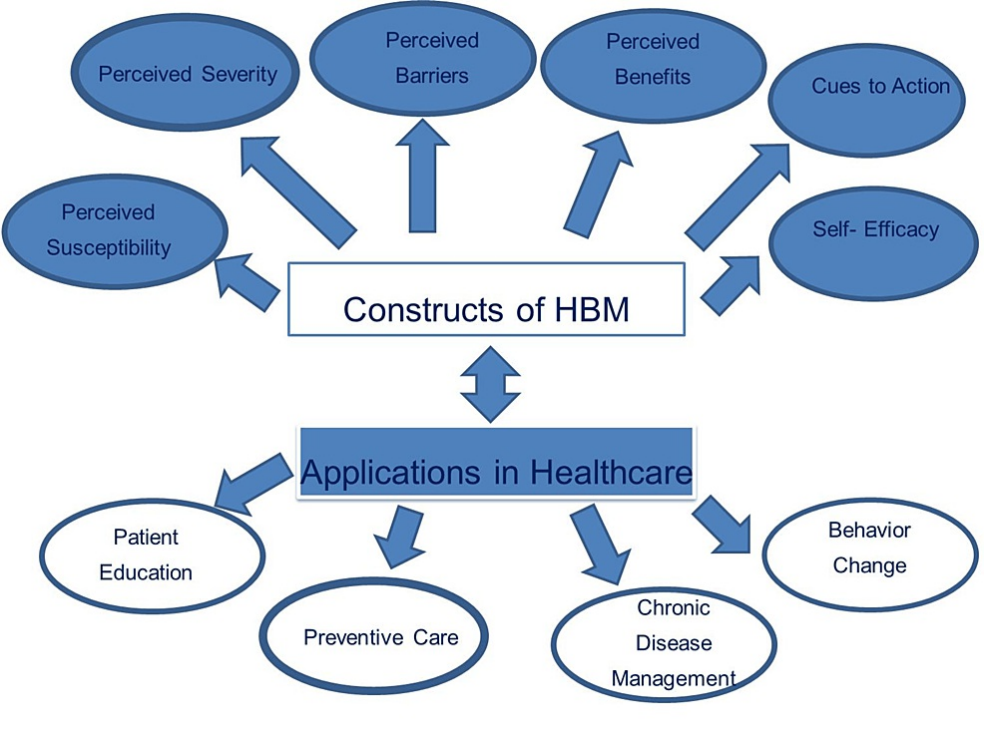
Constructs and applications of HBM. [27-30]

The main objective of this study is to conduct a comprehensive scientometric exploration of behavior change theories and models within the context of the HBM (Figure 6) over the past five decades. The goal is to provide valuable insights into the intellectual structure, thematic evolution, and emerging research directions of this field to inform future research priorities, interdisciplinary collaborations, and the development of more integrated theoretical approaches to the change of health behavior.

Lau JTF, a prominent researcher affiliated with the Chinese University of Hong Kong, has made significant contributions to the field of HBM research through his extensive work on a wide range of public health topics. His studies have focused on preventive behaviors and responses to mental distress to outbreaks of infectious diseases, such as the H1N1 pandemic, among university students in China [11]. He has also conducted longitudinal research on the incidence and predictive factors of Internet addiction among secondary school students in Hong Kong, utilizing the HBM framework [31]. Furthermore, his work has examined the coverage and parental perceptions of influenza vaccination and the barriers and factors associated with colorectal cancer screening behavior in Chinese populations [32,33]. Lau's research has further explored the parental acceptability of HPV vaccination and, more recently, the acceptance and intention of the COVID-19 vaccine among the general public and healthcare workers in Hong Kong [13,28,34-37]. This diverse range of topics covered in his research underscores the depth of his understanding and the versatility of HBM in understanding health-related decision-making processes.

The data from the publication in Figure 2 reveal a steady and accelerating growth in HBM research in recent decades. From a low base of 1-35 articles annually in the early years (1974-1989), HBM publications saw a pickup in the 1990s and then a surge from the 2000s onward, consistently exceeding 100 articles per year and reaching a peak of 671 (11.42%) in 2022. The significant increase in HBM publications in recent decades can be attributed to factors such as the growing recognition and versatility of the model, advancements in research methodologies, increased funding opportunities, and the availability of publishing platforms. These factors have collectively contributed to the sustained expansion of HBM research, indicating its enduring relevance and utility in studying health behavior. This sustained increase in scholarly focus on HBM aligns with and extends previous observations, indicating the model's growing prominence and versatility in understanding and promoting various health behaviors [9,38,39]. The continued expansion of HBM research suggests its enduring relevance and utility in research and practice on health behavior.

The United States' dominance in HBM research stems from its robust research infrastructure, funding, collaborative academic environment, focus on population health challenges, global influence, and the historical legacy of the model's development within the country [21,40]. The combination of these factors has contributed to the United States' dominant position in HBM research, enabling its researchers to drive the development, application, and advancement of HBM across a wide range of health behavior domains.

Keyword analysis suggests that the HBM theme (Figure 3B) is a central and well-developed theoretical framework in research related to health behaviors, particularly in the context of the COVID-19 pandemic and other infectious diseases. The theme showed that HBM is a foundation for understanding and predicting various health-related behaviors, such as vaccination, preventive behaviors, and health practices, in various populations, including adolescents, pregnant women, older adults, college students, and the general public [13,32,33,35-38]. Research encompasses both individual-level factors and broader public health and health communication aspects, utilizing both quantitative and qualitative approaches and frequently integrating HBM with other theoretical frameworks, such as the Theory of Planned Behavior and Social Cognitive Theory [1,23]. The broad applicability of HBM extends beyond infectious diseases, encompassing other health domains, such as oral health and human papillomavirus (HPV) vaccination, highlighting the versatility of this well-established framework and ongoing efforts to understand and promote health-enhancing behaviors using this theoretical foundation [35,39].

Thematic mapping indicates that breast cancer and related health behaviors, such as breast self-examination and mammography screening, are niche themes within the broader research landscape [41]. Although specialized, these topics appear to be areas of ongoing investigation, focusing on examining the validity and reliability of the measures and constructs associated with HBM in the context of breast cancer prevention and early detection [12,42]. The research likely explores the role of perceived susceptibility, perceived severity, perceived benefits, and other HBM constructs in shaping women's attitudes, intentions, and behaviors regarding breast self-examination and participation in mammography screening programs [12,41,42]. This specialized line of inquiry contributes to a deeper understanding of the psychological and social factors that influence breast cancer-related health practices, which is crucial for the development of more effective health promotion and early detection strategies targeting this critical public health issue [30].

The theme of "health behavior" (Figure 3B) is identified as a basic and foundational aspect within the research landscape that utilizes HBM, indicating the widespread application of the model to understand, predict, and promote a diverse array of health behaviors in various health conditions and populations. Keywords associated with this theme encompass a wide range of health-related actions, including adherence and compliance to medical treatments, preventive and screening behaviors, self-management of chronic conditions, and health promotion and prevention of diseases. Researchers in this domain employ a variety of methodological approaches, such as qualitative research, systematic reviews, and the use of mobile health technologies, to gain a comprehensive understanding of the complex factors influencing health behaviors [1,14,15,22,24,37,42]. The centrality of the theme "health behavior" underscores the versatility and relevance of HBM in examining a wide range of health-related practices, which is crucial for the development and implementation of targeted health interventions, patient education programs, and health promotion strategies aimed at addressing various public health challenges and health disparities, often through the integration of HBM with other theoretical frameworks [29]. The COVID-19 pandemic has presented a unique and urgent set of health challenges, leading researchers to direct their attention toward understanding the factors influencing health behaviors during this crisis. The theme of COVID-19 has gained prominence as researchers seek to understand and promote desirable health behaviors related to preventive measures, vaccination, and adherence to public health guidelines. Within the context of the HBM, constructs such as perceived susceptibility and severity of the disease, cues to action (such as public health messaging), and perceived benefits and barriers to preventive behaviors provide a valuable framework for understanding and guiding behavior change efforts during the pandemic [2,8,19,27,36,37].

The rapid advancement of technology has opened new avenues for health behavior research, particularly in the realm of mobile health (mHealth) interventions. The theme of mHealth focuses on utilizing mobile devices and applications to deliver health-related information, interventions, and support. This emerging theme aligns with several core constructs of the HBM. For instance, cues to action can be provided through mobile notifications and reminders, self-efficacy can be enhanced by providing individuals with tools and resources through mobile apps, and perceived benefits can arise from the convenience and accessibility of technology for health management. By incorporating the constructs of the HBM, researchers can effectively study and promote behavior change within the context of mHealth interventions [1,14,15,22,24,37,42].

Despite the well-established success of vaccination as a public health intervention, a growing number of people now view vaccines as unsafe and unnecessary. This lack of confidence in vaccines is seen as a threat to the effectiveness of vaccination programs. Vaccine hesitancy is believed to be a contributing factor behind declining vaccine coverage rates, which, in turn, increases the risk of outbreaks and epidemics of vaccine-preventable diseases [43]. Vaccine hesitancy is a trending topic (Figure 5) in HBM research, as a growing number of individuals perceive vaccines as unsafe and unnecessary, leading to decreased vaccine confidence and coverage. Applying HBM, researchers are examining how factors such as perceived susceptibility, severity, benefits, barriers, and cues to action shape vaccine-related beliefs and behaviors [2,8,27,44,45]. This research aims to identify key determinants of vaccine hesitancy to inform targeted interventions and communication strategies, as maintaining high vaccination rates is crucial for population health and preventing the resurgence of vaccine-preventable diseases [2,45].

The bibliometric analysis of HBM research has several limitations, including a limited scope of only Scopus-indexed publications, potential biases in citation-based metrics, thematic analysis constraints, lack of contextual factors, and limited generalizability of the global findings. These limitations should be taken into account when interpreting the insights and understanding the broader HBM research landscape.

## Conclusions

The HBM has seen a surge in scholarly attention over five decades. Publications are steadily increasing, peaking at more than 11% in 2022-2023. Research is global, with the United States, Iran, China, the UK and Australia leading. Top researchers such as Lau, Champion, and Jeihooni contribute influential work, and collaborations are the most robust between these countries. Highly cited HBM publications showcase the model's enduring relevance across health behaviors. Thematic analysis reveals a shift from broader topics of HBM to specific areas such as COVID-19, health behavior, and digital health interventions, reflecting evolving research priorities. This bibliometric analysis highlights the sustained interest in HBM, its diverse applications, and global collaboration to refine this influential public health and behavioral science framework.

Recommendations

The HBM provides a valuable framework for understanding health behaviors, but research gaps still need to be addressed. Although adept at explaining cognitive factors, HBM must fully account for individual differences in personality, emotions, or habitual behaviors. It also needs to better address the significant impact of social determinants and cultural contexts on health decisions. Future research directions include refining HBM to incorporate social and emotional factors, exploring its application in diverse populations, and evaluating its effectiveness in promoting specific behaviors across contexts. Some propose integrating HBM with other models for a more comprehensive understanding of health decision-making.
